# Directed and target focused multi‐sectoral adolescent HIV prevention: Insights from implementation of the ‘DREAMS Partnership’ in rural South Africa

**DOI:** 10.1002/jia2.25575

**Published:** 2020-08-31

**Authors:** Natsayi Chimbindi, Isolde Birdthistle, Sian Floyd, Guy Harling, Nondumiso Mthiyane, Thembelihle Zuma, James R Hargreaves, Janet Seeley, Maryam Shahmanesh

**Affiliations:** ^1^ Africa Health Research Institute Mtubatuba South Africa; ^2^ London School of Hygiene and Tropical Medicine London United Kingdom; ^3^ Institute for Global Health University College London London United Kingdom; ^4^ Harvard University Boston MA USA; ^5^ University of the Witwatersrand Johannesburg South Africa

**Keywords:** adolescent girls and young women, HIV, holistic, interventions, layering, implementation

## Abstract

**Introduction:**

The “DREAMS Partnership” promotes a multi‐sectoral approach to reduce adolescent girls and young women's (AGYW) vulnerability to HIV in sub‐Saharan Africa. Despite widespread calls to combine structural, behavioural and biomedical HIV prevention interventions, this has not been delivered at scale. In this commentary, we reflect on the two‐year rollout of DREAMS in a high HIV incidence, rural and poor community in northern KwaZulu‐Natal, South Africa to critically appraise the capacity for a centrally co‐ordinated and AGYW‐focused approach to combination HIV prevention to support sustainable development for adolescents.

**Discussion:**

DREAMS employed a directed target‐focused approach in which local implementing partners were resourced to deliver defined packages to AGYW in selected geographical areas over two years. We argue that this approach, with high‐level oversight by government and funders, enabled the rapid roll‐out of ambitious multi‐sectoral HIV prevention for AGYW. It was most successful at delivering multiple interventions for AGYW when it built on existing infrastructure and competencies, and/or allocated resources to address existing youth development concerns of the community. The approach would have been strengthened if it had included a mechanism to solicit and then respond to the concerns of young women, for example gender‐related norms and how young women experience their sexuality, and if this listening was supported by versatility to adapt to the social context. In a context of high HIV vulnerability across all adolescents and youth, an over‐emphasis on targeting specific groups, whether geographically or by risk profile, may have hampered acceptability and reach of the intervention. Absence of meaningful engagement of AGYW in the development, delivery and leadership of the intervention was a lost opportunity to achieve sustainable development goals among young people and shift gender‐norms.

**Conclusions:**

Centrally directed and target‐focused scale‐up of defined packages of HIV prevention across sectors was largely successful in reaching AGYW in this rural South African setting rapidly. However, to achieve sustainable and successful long‐term youth development and transformation of gender‐norms there is a need for greater adaptability, economic empowerment and meaningful engagement of AGYW in the development and delivery of interventions. Achieving this will require sustained commitment from government and funders.

## 1 INTRODUCTION

In South Africa HIV incidence remains high, especially among adolescents and youth (10 to 25 years old) [1]. Although there is evidence of a decline in HIV incidence of 44% among the general population from 2012, incidence was still higher in adolescent girls and young women (AGYW) [15, 16, 17, 18, 19, 20, 21, 22, 23, 24] than their male counterparts [2]. This indicates there is still need for greater efforts to reduce the impact of the HIV epidemic in young people, in particular AGYW [1].

**Figure 1:**
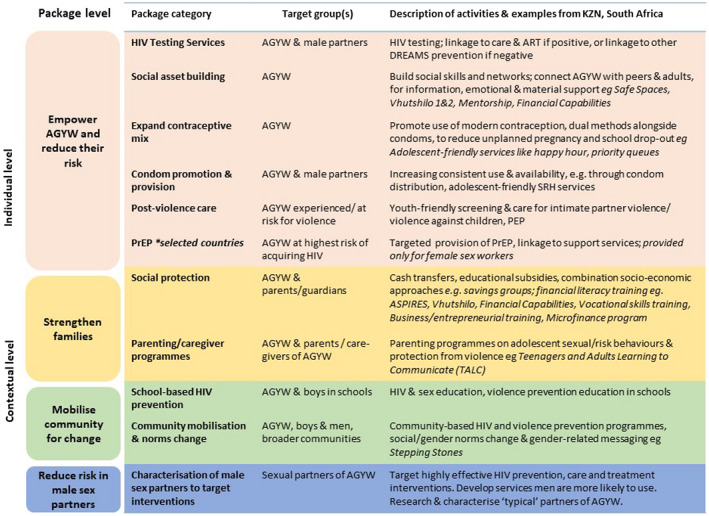
Framework for DREAMS core package of interventions. Adapted with permission [12].

There have long been calls to scale‐up evidence‐based combination structural, behavioural and biomedical HIV prevention interventions [3, 4, 5, 6, 7]. This has been reinvigorated by evidence that “layering,” that is providing multiple interventions or services can accelerate progress to sustainable development goals in adolescents living with HIV [8]. In response, the US Presidents’ Emergency Plan for AIDS Relief (PEPFAR) with others, supported the ‘DREAMS Partnership’, a multi‐sectoral package of interventions targeting multiple sources of HIV risk and vulnerability for AGYW [9, 10]. The aim of DREAMS was to reduce HIV incidence through strengthening existing interventions and the introduction of new packages for gender‐based violence, family and caregiving, social asset building, economic empowerment/cash transfers and pre‐exposure prophylaxis (PrEP) (Figure 1) [9, 11, 12].

DREAMS in South Africa was implemented with high‐level oversight by government and funders, through local implementing partners who were resourced to deliver defined and target‐focused packages of interventions to AGYW in selected geographic areas over two years. Implementing multi‐sectoral programmes is complex [3]; it requires maintaining fidelity to the Theory of Change, coordination across multiple sectors and monitoring coverage of those in need [11, 13]. Recognizing these challenges, between 2016 and 2018 we evaluated DREAMS rollout in a poor rural district in northern KwaZulu‐Natal (KZN), South Africa, with a high burden of HIV through extensive engagement with implementing partners, community stakeholders and representative surveys of potential beneficiaries of DREAMS [13].

In this commentary we, a multidisciplinary team of researchers, reflect on our experience to appraise the capacity for a co‐ordinated and AGYW focused approach to combination HIV prevention to support sustainable development for adolescents. We argue that this approach rapidly scaled‐up a multi‐sectoral HIV prevention intervention for AGYW. It was most successful when it strengthened existing infrastructure and/or when tackled youth development that coincided with community concerns. We interrogate and draw lessons from the lost opportunity to support longer term sustainable development goals and transform gender norms for adolescents [14, 15, 16].

## 2 DISCUSSION

### 2.1 Prescribed and target‐driven scale‐up of multi‐sectoral HIV prevention for AGYW

Prior to DREAMS, there was limited co‐ordination of HIV interventions for adolescents and young people in the study area in northern KZN. Health promotion and preventive services were mostly provided through the Department of Health in fixed clinics; life orientation was provided in schools by the Department of Education, and social protection by the Department of Social Development [17]. Prior to 2015, HIV incidence had been persistently high in this area [18] with low uptake of sexual and reproductive health services; in 2015 <50% of sexually active AGYW used condoms at last sex; and <50% were currently using contraception [18].

In order to catalyse multi‐sectoral collaborations and strengthen existing resources and policies, such as government cash transfer mechanisms to support AGYW [6] and adolescent and youth friendly services [19], the DREAMS Partnership engaged with the Departments of Health, Social Development and Education [11, 20]. However, the rollout of DREAMS was very rapid [20], and these sectors had not previously been co‐ordinated by a disease‐specific agency such as the AIDS Council. The AIDS council was involved at provincial level as the co‐ordinating body and at district level as part of the project team responsible for co‐ordinating DREAMS and ensuring the alignment of DREAMS activities with existing programmes [20]. Moreover, some of the interventions were new to the setting, such as community‐based interventions for gender‐based violence, family and caregiving and HIV PrEP. Five implementing partners were commissioned, each to deliver different interventions of the package based on their expertise. While some implementing partners subcontracted community‐based organizations (CBOs) who were embedded in the community, others introduced new organizations to the area [20].

The consequence was that at the height of implementation in 2017, 11 organizations were receiving DREAMS funding to deliver 28 different interventions, grouped into categories (e.g. social protection), which in turn were organized by levels (e.g. “strengthen families”) (Figure 1) [12] that were expected to be layered in order to accelerate benefits in AGYW. Layering also included contextual interventions that were not delivered directly to the individual, but benefitted the AGYW [11, 12, 21]. In the absence of an existing co‐ordinating mechanism, several donor‐led steering committee meetings were organized to bring all the players on‐board, mapping out geographical working boundaries and ensuring the “layering” approach was understood [21]. Implementing partners were given targets (number of AGYW to reach with specific interventions), which were monitored through the DREAMS Integrated Monitoring and Evaluation System (DIMES) [22]. Quarterly provincial meetings (and monthly at district‐level) were held by the co‐ordinating partner and AIDS council with implementing partners to measure progress and performance.

In the next section, we appraise the strengths and weaknesses of this prescribed and target‐driven approach to combination HIV prevention for AGYW in supporting sustainable development goals among youth in a rural community.

### 2.2 Lessons learned

#### 2.2.1 Effective scale‐up strengthens existing infrastructure and builds on intervention norms

Interventions that built on pre‐existing interventions with organizations that were already embedded in the setting could be scaled‐up rapidly, for example school‐based interventions, HIV testing, condom distribution and promotion through existing CBOs, since the infrastructure already existed and little training and adaptation were needed.

CBOs who were already embedded in the communities were able to adapt delivery (but not content) of the DREAMS package to the local context. For example they offered HIV testing during outreach activities at community gatherings (grants pay‐out days, sport days, etc), distributed condoms in shops in the rural settings and formed partnerships with private doctors, police and other implementing partners to support identification and management of post‐violence care and improved onward referrals. As a result we found an increased visibility of these CBOs and their activities, such as condoms in bars, shops and remote rural areas.

Some novel interventions such as voluntary medical male circumcision (delivered outside of DREAMS, but escalated during DREAMS) that responded to and resonated with the existing HIV prevention and gendered norms, such as traditional male circumcision, were acceptable and uptake increased [23]. However, while more young people reported being aware of newer biomedical technologies such as PrEP by the second year of scale‐up, they and healthcare workers expressed ambiguous feelings around this novel biomedical approach to HIV prevention [23, 24]. Young people were concerned about side effects related to the use of PrEP and the potential HIV‐related stigma and discrimination they could experience if as young women they accessed PrEP from healthcare facilities [23, 25].

#### 2.2.2 Youth development was embraced by the community; transforming gender‐norms less so

Unemployment, poverty and violence are recognized as youth development issues of importance in the area, and therefore community members welcomed the broader multi‐sectoral approach that underpinned DREAMS. This was particularly the case when delivered through CBOs with a history in the area, which were trusted and embraced the benefits of “layering” interventions. Consequently, there was a rapid increase in the proportion of AGYW who received all three “layers” of DREAMS interventions, that is interventions at community, family and individual levels (Figure 1). More than half of AGYW were invited to participate in DREAMS, with over 80% of those accessing ≥3 interventions [12].

Community leaders saw DREAMS multi‐sectoral approach as a lost opportunity to include young men who faced similar youth development challenges [26]. While young men have sexual reproductive health (SRH) needs and are partners of AGYW [26] in our community this ambivalence mirrored the well‐described barriers to shifting gender‐norms in South Africa [27, 28, 29]. DREAMS implemented a package that addressed gender‐based violence explicitly and gender dynamics implicitly (contraception education and access, stepping stones and cash transfers). However, the prescribed nature of the packages and limited opportunities for meaningful engagement of young women and men in implementation, constrained the transformative potential to radically challenge social constructs of gender that continue to drive the disproportionate burden of HIV on adolescents and young women [30, 31, 32].

#### 2.2.3 Youth centred adaptation to social context is an important ingredient

DREAMS implementing partners were required to deliver interventions listed in the DREAMS package as per their contractual agreement and area of expertise and were monitored with respect to centrally designed standards of delivery. Organizations delivering these interventions felt that they could be more successful if they were able to adapt to their social context and respond to unmet youth development needs. However, they felt a tension between this and being seen to deliver interventions with fidelity to the central design standards. The overall effect was a limited scope for iterative adaptation or innovation.

Even after DREAMS rollout, contraception uptake remained low among adolescent girls [13, 14, 15, 16, 17, 18, 19] despite many being sexually active [12, 33]. We found that strengthening provision of adolescent and youth‐friendly SRH services within the primary healthcare clinics in this rural setting during the period of evaluation did not translate to uptake; well‐described social, health facility and individual level factors all contributed to poor uptake. At an individual level, persisting myths and misconceptions around conception [23, 33] and anticipated stigma associated with being seen entering a clinic, fear of judgement and transport costs were described by AGYW as barriers to use. Data from our team suggested that young people and the organizations working with them felt that more active involvement of young people may have increased demand for services and promoted innovations in healthcare delivery that overcome barriers to uptake; for example the use of peer outreach workers to promote sexual health and delivery of SRH services in youth centres and mobile clinic [31, 34, 35].

Similarly, there was limited flexibility within DREAMS to respond to other health issues such as mental health and alcohol use, even though they are well‐described [36] structural factors that predispose young people to HIV acquisition and poor health. Common mental health disorders increased steadily with age among AGYW in this setting (up to 33% in 22‐year olds), and were associated with food insecurity, migration and experiencing violence [36]. Similarly, alcohol was easily available to and perceived as a normative part of adolescence and transition into adulthood. Poor mental health and alcohol were described as barriers to engagement and retention in the prevention, treatment and care services offered by DREAMS. For example young people described engaging in unplanned and unprotected sex under the influence of alcohol or drugs and reported forgetting to take their PrEP or ART pills when drunk [23, 24, 37].

#### 2.2.4 Target focused delivery may reduce reach to those most in need

In a setting where there were few prior HIV interventions targeting young people, local implementing partners had to develop new ways to identify vulnerable AGYW to reach. They relied on their organizational databases of orphans and vulnerable children and families and worked with schools for recruiting and targeting AGYW in need of services. This targeting of DREAMS interventions by place or type of person, with goal of “saturating” targeted AGYW may have, particularly under the pressure to rapidly implement, paradoxically hindered reach to those most in‐need or vulnerable.

During multiple donor‐led meetings and with resources focused on geographical mapping and identifying higher risk AGYW, the challenges local implementers faced became apparent. Vulnerable AGYW were widely dispersed, often mobile and engaging AGYW in the geographical areas where HIV‐infection was high, was frequently a challenge. For example we found more than one in ten of sexually active AGYW reported transactional sex or sex‐work activities, but only a handful of them were aware of PrEP and none had taken PrEP, a service that was specifically targeted at this group of young women [24]. AGYW engaging in commercial sex often did not self‐identify or report themselves as sex‐workers and were thus missed by PrEP outreach programmes [24]. A differentiated approach, investing in universal health and social services for adolescents and young people that could be tailored to individual needs, combined with evidence‐based approaches to reaching those who are harder to reach, such as through social networks or venue‐based approaches, may result in more effective coverage of vulnerable and at risk AGYW in this type of rural setting [17].

#### 2.2.5 Youth leadership and sustainable development goals

DREAMS was a lost opportunity to embed sustainable development goals and build the capacity for youth leadership in a deprived rural community. Youth unemployment was high (>80% among 18+ year‐olds) [18] and there was a lack of recreation and educational opportunities for young people who had completed school, increasing vulnerability to transactional sex and crime [37]. Migration was high among this group (about 20% among AGYW in 2017 reported ever migrating in the past year) mainly for seeking employment and school purposes, and these AGYW were missed out of interventions [12], yet they are at high risk [38].

While DREAMS did support the delivery of many of the development accelerators such as government cash transfers [39], support to stay in schools, parenting support and safe spaces, there was limited investment in long‐term interventions to strengthen employability and income generation, such as skill building or microfinance initiatives [6, 30]. Furthermore, there was little done to build youth capacity to deliver these or actively engage in the local DREAMS co‐ordination mechanism [31]. The transition out of the DREAMS Partnership in the study area, after two years, happened shortly after the implementing partners had gained traction and started to implement this complex intervention. The absence of local leadership and in particular youth leadership left a void in co‐ordinating the multiple sectors with no‐one to actively advocate for sustaining activities post‐DREAMS funding, and ensure the capacity and skills gained during DREAMS could be useful for the CBOs activities post‐DREAMS [16, 40, 41].

## 3 CONCLUSIONS

Centrally directed, prescribed and target‐focused scale‐up of multi‐sectoral HIV prevention interventions for AGYW in a poor rural South African setting was largely successful in rapidly reaching AGYW and layering development accelerators such as government cash transfers, parenting support, violence interventions, safe spaces and friendly health services for AGYW. The approach was most successful when it built on the capacity of existing infrastructure and brought resources to tackle youth development of concern to the community. However, to protect young people better and achieve sustainable and successful long term youth development, we need greater adaptability and meaningful engagement of AGYW in the development and delivery of the intervention [14, 15, 16]. Expanding holistic HIV prevention interventions such as the DREAMS partnership to support youth development, including economic empowerment, and mobilizing youth to transform gender norms, and build social capital may provide the foundation for a sustained impact on the HIV epidemic and improvements in the wellbeing of young people in sub‐Saharan Africa. Achieving this will require sustained commitment from government and funders.

## COMPETING INTERESTS

The authors declare they have no conflict of interest.

## AUTHORS’ CONTRIBUTIONS

Natsayi Chimbindi, Maryam Shahmanesh and Isolde Birdthistle involved in conceptualization. Natsayi Chimbindi and Maryam Shahmanesh also involved in writing the original draft of the manuscript. Maryam Shahmanesh, Natsayi Chimbindi, Isolde Birdthistle, Guy Harling, James Hargreaves, Nondumiso Mthiyane, Sian Floyd, Thembelihle Zuma, Janet Seeley and Sian Floyd contributed to reviewing and editing of the manuscript. All authors reviewed drafts and read and approved the final manuscript.
